# Dietary Lipids Influence Bioaccessibility of Polyphenols from Black Carrots and Affect Microbial Diversity under Simulated Gastrointestinal Digestion

**DOI:** 10.3390/antiox9080762

**Published:** 2020-08-17

**Authors:** Chunhe Gu, Hafiz A. R. Suleria, Frank R. Dunshea, Kate Howell

**Affiliations:** 1 School of Agriculture and Food, Faculty of Veterinary and Agricultural Sciences, The University of Melbourne, Parkville 3010, VIC, Australia; chunheg@student.unimelb.edu.au (C.G.); hafiz.suleria@unimelb.edu.au (H.A.R.S.); fdunshea@unimelb.edu.au (F.R.D.); 2 Faculty of Biological Sciences, The University of Leeds, Leeds LS2 9JT, UK

**Keywords:** anthocyanin, phenolic acid, antioxidant capacity, medium chain fatty acids, short chain fatty acids, gut microbiome

## Abstract

The bioaccessibility and activity of polyphenols is dependent on their structure and entrapment in the food matrix. While dietary lipids are known to transit into the colon, the impact of different lipids on the microbiome, and their interactions with dietary polyphenols are largely unknown. Here, we investigated the effect of dietary lipids on the bioaccessibility of polyphenols from purple/black carrots and adaptation of the gut microbiome in a simulated in vitro digestion-fermentation. Coconut oil, sunflower oil, and beef tallow were selected to represent common dietary sources of medium-chain fatty acids (MCFAs), long-chain polyunsaturated fatty acids (PUFAs), and long-chain polysaturated fatty acids (SFAs), respectively. All lipids promoted the bioaccessibility of both anthocyanins and phenolic acids during intestinal digestion with coconut oil exhibiting the greatest protection of anthocyanins. Similar trends were shown in antioxidant assays (2,2-Diphenyl-1-pricrylhydrazyl (DPPH), ferric reducing ability (FRAP), and total phenolic content (TPC)) with higher phytochemical bioactivities observed with the addition of dietary lipids. Most bioactive polyphenols were decomposed during colonic fermentation. Black carrot modulated diversity and composition of a simulated gut microbiome. Dramatic shifts in gut microbiome were caused by coconut oil. Inclusion of sunflower oil improved the production of butyrate, potentially due to the presence of PUFAs. The results show that the impact of polyphenols in the digestive tract should be considered in the context of other components of the diet, particularly lipids.

## 1. Introduction

Polyphenols are naturally occurring compounds found in fruits and vegetables, and have been widely studied as beneficial compounds for health based on their antioxidant activity. In the human body, absorbed polyphenols prevent the generation of free radicals and reactive oxygen species (ROS) and, therefore, increase the plasma antioxidant defences [1,2]. Cardiovascular diseases are related with excessive absorption of triglycerides and triglyceride-rich lipoproteins [3]. Absorbed polyphenols can prevent accumulation of oxidised plasma triglycerides and lipoproteins [4]. Therefore, bioavailable polyphenols can potentially mitigate the adverse effects of high levels of plasma triglycerides, and are considered beneficial health compounds which can lower the risks of cardiovascular disease in humans. After consumption, polyphenols are released from the food matrix in the stomach and small intestine and are absorbed by intestinal enterocytes [5]. However, most polyphenols bound to the food matrix escape small intestinal digestion to be transported to the colon for fermentation and further metabolism by the gut microbiota [6]. Therefore, bioaccessibility of polyphenols released at different digestive stages as well as the interactions of polyphenols with macronutrients of the food matrix and gut microbiota are crucial factors for understanding polyphenol bioavailability.

High plasma triglycerides concentrations may be a result of excessive dietary lipid consumption [7]. Although lipids are absorbed in the small intestine, some dietary fats escape absorption and reach the colon, leading to structure shifts in the gut microbiome [8]. Fats in the food matrix increase the bioaccessibility of polyphenols during digestion. Ortega, et al. [9] reported an increased recovery of phenolic compounds from cocoa during an in vitro duodenal digestion and additionally that poly-unsaturated fatty acids (PUFAs) released from the food matrix during digestion enhanced the stability of phenols from a carob flour [9]. Shishikura, et al. [10] verified that tea polyphenols could interact with olive oil and shifted the extent of oil emulsification during digestion. However, the effects of the type of lipids on bioaccessibility of polyphenols during digestion as well as the reciprocal interactions between a polyphenol-lipid matrix and gut microbiota remain largely unclear.

In this study, a standardised static in vitro digestive system was used. Black carrots (*Daucus carota ssp. sativus var. atrorubens Alef.*), which are rich in anthocyanins and phenolic acids [11], were used as a source of polyphenols. Coconut oil, sunflower oil, and beef tallow were used to represent different dietary sources of medium-chain fatty acids (MCFAs), longer chain PUFAs, and long-chain polysaturated fatty acids (SFAs), respectively (Table 1). Antioxidant activity and bioaccessibility of individual black carrot polyphenols with the presence of fats in gastric, small intestinal and colonic digestive stages were measured. The effects of different polyphenol-fat combinations on a fermentation of a gut microbiome was investigated. The present study unravels the role of lipid in the polyphenol digestion-fermentation process and the corresponding shift in microbial diversity and metabolites production in a gut fermentation.

## 2. Materials and Methods

### 2.1. Chemicals and Reagents

All chemicals were analytical grade or analytical standards and were purchased from Sigma-Aldrich (Castle Hill, NSW, Australia), unless stated. Potassium chloride, potassium dihydrogen phosphate, sodium hydrogen carbonate, sodium chloride, magnesium dichloride, ammonium carbonate, sodium hydroxide, hydrogen chloride and calcium chloride were used for the digestive electrolyte stock solution preparation. Pepsin from porcine gastric mucosa, pancreatin from porcine pancreas, and porcine bile extract were the enzymes used for simulating gastric and small intestinal digestions. Pancreatin from porcine pancreas was purchased from US Biological (Assay Matrix Pty Ltd., Australia). Starch, peptone, tryptone, yeast extract, pectin, mucin, casein, L-Cysteine hydrochloride anhydrous, magnesium sulphate, guar, dipotassium phosphate, bile salts, and Tween-80 were used for the colonic basal media preparation. Analytical grade methanol, ethanol and formic acid were used for polyphenols extraction. Folin-Ciocalteu reagent, sodium carbonate, gallic acid, 2,2-Diphenyl-1-pricrylhydrazyl (DPPH), ascorbic acid, sodium acetate, 2,4,6-tripytidyl-s-triazine (TPTZ), and ferric chloride were used for the antioxidant assays. The 96-well plates (flat bottoms with 300 µL total volume) (Corning Inc., Corning, NY, USA) were purchased from Thermo Fisher Scientific (Scoresby, Australia). HPLC analytical grade chlorogenic acid, caffeic acid, *p*-coumaric acid, ferulic acid, cyanidin-3-*O*-glucoside, acetic acid and acetonitrile were for chromatographic analysis. The 1.5 mL HPLC vials were purchased from Agilent Technologies (Mulgrave, Australia).

### 2.2. Sample Preparation

Black carrots (*Daucus carota sativus var. atrorubens*) and commercial oils, including sunflower oil, coconut oil, and beef tallow were purchased from a local market in Melbourne, Victoria. Black carrots were grown in Melbourne, Victoria. About 300 g carrots were peeled and weighed, followed by being chopped into 1 cm^3^ cubes, which were then put into a mortar and instantly frozen in liquid nitrogen. The frozen carrot cubes were then ground into fine powder using a coffee grinder (Sunbeam^®^ Multi Grinder—EM0405) and lyophilised to constant weight. The powder was then weighed and a dry/wet weight ratio of 0.1 was gathered. Afterwards, aliquots of 0.2 g carrot powder were weighed in a 50 mL centrifuge tube. When the lipid preparations were included, tallow and coconut oil were melted by heating at 40 °C, and then 1 g fat of each type was weighed and mixed with the carrot powder to make combinations of black carrot with coconut oil (BC), sunflower oil (BS), and tallow (BT) samples. The setting of carrot/fat ratio was according to the dietary guidelines recommended by the National Health and Medical Research Council of Australian Government (2015), where the ratio of vegetables to other meal components containing fat was about 0.2. The percentage of the fat content of the basal diet was estimated as 10% by weight basis. Therefore, the ratio of dry carrot powder/fat was approximate 0.2. Controls with black carrot only (0.2 g) (B) and fat only (1 g) of coconut oil (C), sunflower oil (S) and tallow (T) were also prepared. The samples were then homogenised at 5000 rpm for 2 min using an IKA Ultra-Turrax^®^ T25 homogenizer (Rawang, Selangor, Malaysia) ready for the in vitro digestive treatments. For extraction of polyphenols from carrots, the frozen carrot powder was extracted using a solvent of methanol/formic acid/water (80:1:19, *v/v/v*) at a ratio of 50 mg/mL. The extraction was taken in a shaking incubator (ZWYR-240, Labwit, Ashwood, Australia) at 120 rpm, 4 °C for 10 h in darkness followed by centrifugation at 10,000× *g* for 10 min. The supernatant was collected for instrumental analysis.

### 2.3. In Vitro Gastrointestinal Digestion

Samples were subjected to simulated in vitro gastro-intestinal digestive systems described by Pérez-Burillo et al. [13]. Briefly, the gastro-intestinal digestion was composed of gastric phase (2 h at 37 °C with pepsin 2000 U/mL, pH 3.0) and small intestinal phase (2 h at 37 °C with pancreatin 13.37 mg/mL, pH 7.0). All procedures were carried out in a shaking incubator at 100 rpm in darkness. The reactions were terminated by instantly freezing the samples in liquid nitrogen which were then stored in −80 °C for further analysis.

### 2.4. In Vitro Colonic Fermentation

The in vitro colonic fermentation procedure was carried out according to the protocol of Fu, et al. [14] with modifications. Pig faeces were used as sources of gut microbiome as a substitute of human faeces, as pigs and humans are primarily colonic fermenters sharing a comparable gut microbiome [15]. Five female large White × landrace grower pigs with liveweight of about 50 kg and raised in animal house of University of Melbourne. The selected pigs were fed with standard grower diet for two weeks. The faeces were taken immediately after the pigs defecated, and were put into an anaerobic chamber. The faeces were mixed together, then 20 g of the pooled faeces was weighed, added to 80 g sterilised pre-nitrogen flushed 0.1 M phosphate buffer (pH 7.0) and homogenised for 5 min in a stomacher mixer (MiniMix^®^ Lab Blender, Thomas Scientific, Swedenborg, NJ, USA) to make 20% faecal slurry (*w:w*). The homogenised mixture was filtered through sterile muslin cloth to remove particulate matter. Sediments from the small intestinal digestion were prepared after centrifugation at 10,000× *g* for 10 min. To test the effects of the partial undigested lipids on gut microbiome, 10% of supernatant after small intestine treatments was added to the sediments, as physiologically, 10% of the soluble fraction during digestion enters the large intestine [13]. Afterwards, aliquots of 5 mL faecal slurry were added into the tubes with the sediments followed by adding 5 mL of the basal media. All tubes were flushed with nitrogen and then incubated with shaking at 100 rpm for 20 h in darkness. The tubes were centrifuged at 10,000× *g*, 5 °C for 10 min and the supernatant was taken from the sediment. Supernatants were stored in −80 °C for HPLC and phytochemical bioactivity analyses, and faecal sediments were ready for DNA extraction.

### 2.5. Phytochemical and Antioxidant Assays

The antioxidant potential of the supernatant from each digestive compartment was measured according to a standardised high throughput 96-well plate method [16]. The supernatants were centrifuged at 10,000× *g* for 20 min and 50 µL from the aqueous layer was taken and diluted 10-fold with methanol. Total phenolic content (TPC), 2,2-Diphenyl-1-pricrylhydrazyl (DPPH), and ferric reducing ability (FRAP) were measured to describe the antioxidant potential of the bioaccessible portions of the digesta in stomach, small intestine, and colon. For the fat-oil combined samples, the results were calculated with eliminations of the fat controls.

### 2.6. Separation and Analysis of Polyphenols

Aliquots of 4 mL of the supernatant from the aqueous layer of each digestive compartment after centrifugation (at 10,000× *g* for 20 min) were taken and freeze dried for 24 h, and the solids were added with 1 mL extraction solution (methanol/formic acid/water, 80:1:19, *v/v/v*). Shaking incubated 120 rpm, 4 °C for 10 h in darkness. The extracts were then centrifuged at 10,000× *g* for 5 min. Afterwards, 0.5 mL hexane was added to remove the residual micellised lipids to guard against column clogging. The mixture was left to stand for 2 min until the solvent was separated into two layers. Here, 0.5 mL was taken from the methanol-phenol layer and stored before ready LC separation and analysis.

Separation and quantification of individual polyphenols were performed on Agilent 1200 series HPLC (Agilent Technologies, Santa Clara, CA, USA) equipped with a diode array detector (DAD). A Synergi Hydro-RP (250 × 4.6 mm i.d.) reverse phase column with a particle size of 4 µm (Phenomenex, Lane Cove, NSW, Australia) was protected by a Phenomenex 4.0 × 2.0 mm i.d. C18 ODS guard column. Instrument control, data acquisition and processing were performed using MassHunter workstation software (Qualitative Analysis, version B.03.01, Agilent).

Anthocyanin quantification followed the method of Padayachee, et al. [17] with some modifications using a wavelength of 520 nm. The mobile phase consisted of water/formic acid/acetonitrile (87:10:3, *v/v/v*; eluent A) and acetonitrile/formic acid/water (50:10:40, *v/v/v*; eluent B). The gradient profile was isocratic 10% B (0–5 min), 10-16% B (5–8 min), 16–23% B (8–19 min), 23–100% B (19–22 min), isocratic 100% B (22–27 min), 100–10% B (27–30 min). The flow rate was 0.5 mL/min, and 20 μL was injected for each sample.

Phenolic acid quantification followed the protocol of Gu, et al. [16] using a wavelength of 320 nm. The mobile phase consisted of water/acetic acid (98:2, v/v; eluent A) and acetonitrile/acetic acid/water (100:1:99, *v/v/v*; eluent B). The gradient profile was 0% B (0–5 min), 10–25% B (5–25 min), 25–35% B (25–35 min), 35–40% B (35–45 min), 40–55% B (45–75 min), 55–80% B (75–80 min), 80–90% B (80–82 min), 90–100% B (82–85 min), 100–0% B (85–88 min), isocratic 0% B (88–90 min). The flow rate was 0.8 mL/min and a volume of 20 μL was injected for each sample.

Peak identification was performed in both negative and positive modes with conditions reported by Gu, et al. [16]. All the standard curves were established with five concentrations with *R*^2^ > 0.9. The standards were tentatively characterized using an Agilent 6520 Accurate-Mass Q-TOF LC-MS (Agilent Technologies, Santa Clara, CA, USA) under the same conditions as the samples. Cyanidin-3-*O*-glucoside was used as the standard for anthocyanins. The calculations of the anthocyanins in samples were according to Chandra, et al. [18] and expressed as an equivalence to the applied external standard.

### 2.7. The 16S rRNA Sequencing and Analysis

DNA was extracted from 0.25 g of the fermented samples using a DNeasy^®^ PowerSoil^®^ kit (QIAGEN GmbH, Hilden, Germany). The V3-V4 regions of 16S rRNA gene was amplified using Bakt 341F primers (forward primer CCTAYGGGRBGCASCAG and reverse primer GGACTACNNGGGTATCTAAT). The procedure of 16S rRNA amplicons preparation followed a standard protocol. High throughput sequencing was performed on the Illumina MiSeq platform by Australian Genome Research Facilities (AGRF, Australia). Paired-ends reads were imported and demultiplexed in QIIME 2 (2019.2) [19]. Sequence quality control was performed using DADA2 pipeline (1.12) [20] in R (software version 3.6.2), where the paired reads were filtered, trimmed and merged, and sequence table was constructed with chimeras removed. Phylogenetic tree generation, alpha and beta diversity analysis were performed using the align-to-tree-mafft-fasttree and core-metrics-phylogenetic pipelines of the plugins in QIIME 2 (2019.1). Taxonomic analysis was performed in QIIME 2 (2019.1) using a trained classifier based on SILVA SSU 138 Ref NR 99 sequences and taxonomy [21].

### 2.8. Short Chain Fatty Acids Analysis

The short chain fatty acids (SCFAs) production of the colonic digesta was assessed according to the protocol described by Gu, et al. [11]. Briefly, the acidified post-fermented faeces samples were extracted with water/formic acid (99:1, *v/v*) and analysed using gas chromatography (7890B Agilent, Santa Clara, USA) equipped with a flame ionisation detector (FID). The column was capillary (SGE BP21, 12 × 0.53 mm internal diameter (ID) with 0.5 μm film thickness, SGE International, Ringwood, VIC, Australia, P/N 054473). Helium was used as carrier gas, and the makeup gas consisted with nitrogen, hydrogen and air. The injection volume was 1 μL. 4-methyl-valeric acid was used as the internal standard. Acetic, propionic and butyric acids were analysed for each sample. All results were expressed as mmol/L.

### 2.9. Statistical Analysis

Different carrot-fat treatments were performed with biological quadruplicate digestion-fermentations. One-way analysis of variance (ANOVA) was performed to test the statistical significance among samples at *p* < 0.05 significance level using Minitab^®^ 18 Statistical software (Minitab Inc., State College, PA, USA). GraphPad Prism version 8.0 and XLSTAT (2019) were used for data visualisation. Statistical analysis of gut microbiome data followed the method of Tian, et al. [22], the *p* value for significance was validated by Bonferroni adjustment and reported as *p* < 0.002 with 28 comparisons among 8 different treatments (the *p* < 0.05 value was divided by 28). Multivariate statistical treatments including principal component analysis (PCA) utilising DPPH, FRAP, TPC, and individual polyphenols from different treatment groups and principal coordinate analysis (PCoA) utilising phylogenetic weighted UniFrac distance of microbial beta diversity, which was gathered in QIIME 2, as a measure of sample dissimilarity were performed and visualised using XLSTAT (2019).

## 3. Results and Discussion

This study investigated the role of interactions between lipids and polyphenols in a simulated digestion. Bioaccessible polyphenols and antioxidant activities (DPPH, FRAP, and TPC) were measured to show the digestion of black carrot at each digestive compartment and the influence from dietary lipids. The 16S rRNA sequencing and SCFAs measurement were performed to illustrate the metabolism status of different carrot-lipid matrix.

### 3.1. Evaluation of Bioaccessibility of Individual Phenolic Compounds and Effects of Lipids in Different Digestive Compartments

Five anthocyanins were separated in raw black carrots and the digestive fluids at different digestive compartments (Table 2 and Figure S1). Individual anthocyanins were identified referencing Padayachee, et al. [17]. All were cyanidins and derivatives as reported by Kammerer et al. [23]. Bioaccessibility was calculated as the ratio of the anthocyanins released to the digestive fluids to the corresponding polyphenols extracted from raw carrots. Table 2 shows that at the gastric stage, bioaccessibility of total anthocyanins was 32.7%. The majority of anthocyanins would not decompose during stomach digestion, as anthocyanins are very stable in the acidic condition of the gastric compartment at pH 3 [24]. The remaining portion (67.3%) are non-bioaccessible bound anthocyanins remaining in the food matrix. This result was comparable with other studies where the available anthocyanins were around 30% during the gastric digestion in a plant matrix [25,26]. In comparison, higher bioaccessibility (54.7%) of total phenolic acids was measured (Table 2). Phenolic acids similarly have higher stability at low pH (pH 3) [27], and may be released from their glycosidically bound forms under the actions of hydrolytic enzymes to result in higher measured bioaccessibility [28].

Limited effects of lipids on polyphenols bioaccessibility were shown at the gastric stage except where tallow (BT) had a significantly (*p* < 0.05) lower availability for both anthocyanins and phenolic acids compared to other treatments (Table 2). This could be a result of the high melting point of tallow (~45 °C), which is higher than the temperature during digestion (37 °C), leading to the mixture solidifying and preventing the carrot powder from contacting the digestive fluids. Therefore, most polyphenols would not be released, as lipids are not digested in stomach. No significant differences were seen in coconut (BC) or sunflower (BS) groups compared to the control (B), indicating the undigested lipids would not affect the release of polyphenols from food matrix during gastric digestion.

A decline of anthocyanin bioaccessibility after small intestine treatments was observed with only 0.98% in black carrot control (B) group released (Table 2). It has been verified that there are major reductions of anthocyanins during the small intestinal digestion [29,30], due to low stability of anthocyanins at pH 7. The flavylium cation of anthocyanin molecules is likely to transform to a colourless chalcone, which is an anthocyanin pseudobase and more stable when pH > 5 [26]. Additionally, partial oxidation and degradation of anthocyanins may also occur and form phenolic acids of the B ring during the small intestinal digestion [31]. For phenolic acids, decrease in neochlorogenic acid and chlorogenic acid contents were observed. Similar results were obtained by Gumienna, et al. [27] and Kim, et al. [29], and due to the partial hydrolysis of chlorogenic acids in the small intestine under the influence of intestinal esterase. On the other hand, increased caffeic acid and ferulic acid contents were generated together with newly detected *p*-coumaric acid. Part of these phenolic acids might be the acyl moieties hydrolysed from the corresponding acylated anthocyanins and chlorogenic acid under the actions of intestinal esterase [32]. However, given the relatively high resistance of acylated anthocyanins to gastrointestinal digestion, the newly generated phenolic acids were likely released from bound phenolic acids associated with the plant cell wall of the black carrots under the digestive conditions [32,33].

Lipids significantly affected bioaccessibility during small intestinal digestion as higher (*p* < 0.05) anthocyanin and phenolic acid contents were found preparations containing lipids (BC, BS and BT) compared to the control (B) (Table 2). This result indicated a protective effect of lipids on bioaccessible polyphenols and was in line with previous studies, where the stability and recovery of polyphenols increased with the presence of fat in the food matrix [9,33]. Lipids are emulsified by bile salts and biliary phosphatidylcholine (PC) and break into micelles under the actions of lipase before they are absorbed [34]. The protective effect on polyphenols could be attributed to molecular phenol-lipid interactions. Hydrophobic interactions can exist between anthocyanins and lipids, resulting in incorporation of anthocyanins into the lipid phase of the micelles which prevents the degradation of anthocyanins [10]. For both anthocyanins and phenolic acids, hydrogen bonds could be formed between the hydroxyl groups of polyphenols and the hydrophilic head of PC/digestive enzymes, which could enhance the stability of polyphenols [35,36].

Among the lipid treatments, coconut oil (BC) showed the highest protective effect of anthocyanins with significantly higher bioaccessibility (*p* < 0.05) than sunflower oil (BS) and tallow (BT) at intestinal stage (Table 2). Sunflower and tallow consist primarily of long-chain fatty acids, contrasting with medium-chain fatty acids (MCFAs) forming the majority of fatty acids in coconut oil. The greater protective effects on polyphenols of medium-chain triglycerides could be possibly due to these triglycerides being digested to a greater degree than longer chain triglycerides, as MCFAs possess a higher dispersibility in the aqueous phase [37]. The free MCFAs digested from triglycerides could migrate into the surrounding aqueous phase rapidly and not inhibit the interfacial lipase reaction, instead, long-chain free fatty acids tend to accumulate at the oil-water interface and inhibit lipase activity [38]. As a result, the higher micellarisation efficiency of MCFAs could favour the formation of hydrogen bonds between polyphenols and the hydrophilic head of emulsifiers surrounding the lipid droplets and thus increased the stability of phenols and their bioaccessibility. No differences in anthocyanin bioaccessibility were observed between BS (rich in PUFA) and BT (rich in SFA). Huo, et al. [39] pointed out that the degree of unsaturation of lipids might have more influence on the in vivo absorption of lipophilic bioactive compounds rather than the lipid micellarisation during digestion. Similar results were observed by Colle, et al. [40] who found no significant effects of the degree of unsaturation of lipids on in vitro transfer of lycopene from the food matrix to mixed micelles. For phenolic acids, no statistical differences between types of lipids could be described to explain the bioaccessibility in the small intestinal (Table 2), which might be related to the higher stability of phenolic acids in slight alkaline environment compared to anthocyanins, making the impacts from polyphenol-lipids interactions relatively less. Similar results were gathered by Ortega, et al. [33], where fat content did not affect the digestibility of and stability of the phenolic acids during in vitro duodenal digestion. 

Most of the target polyphenols were not detected after colonic fermentation except ferulic acids in the BC treatment with 66.7% residual bioaccessibility (Table 2), suggesting the polyphenols were metabolised into other compounds by the colonic microbiota. Indeed, most polyphenols can be metabolised by microbes. For example, anthocyanins can be degraded by *Lactobacillus casei* to phenolic acids as seen by degradation to protocatechuic acid in the colon [41]. Cyanidin dioxaloylhexoside, *p*-coumaric acid, ferulic acid, and syringic acid were also reported as metabolites of anthocyanins in other in vitro colonic digestion studies [29,42,43]. In our study, we did not detect phenolic acids, which were most likely to further decomposed into smaller molecules during the extended 20 h colonic fermentation time. *Escherichia, Lactobacillus,* and *Bifidobacterium* can transform both chlorogenic acid and neochlorogenic acid to caffeic acid after 5 h fermentation [44,45], while further degradation of caffeic acid would occur after 5 h with reduction of the double bond to generate dihydrocaffeic acid and then 3-hydroxylpheylpropionia acid [46]. The absence of ferulic acid and *p*-coumaric acid after 20 h of fermentation in the descending colon has been reported when phenolic acid-rich fruits and vegetables are ingested [29,47]. The measurement of ferulic acid in BC group (Table 2) could be due to the protective effect of coconut oil on anthocyanins during intestinal digestion. As a result, ferulic acid might be produced during colonic fermentation as an important metabolite of anthocyanins [48]. Other than that, ferulic acid in conjugated forms protected by coconut oil during small intestinal stage might also be catabolised and produce ferulic acid during the colonic fermentation [49]. Generally, during the in vitro digestion, inclusion of lipids promoted polyphenols bioaccessibility with coconut oil showed the highest protective effects against polyphenols degradation.

### 3.2. Antioxidant and Phytochemical Assays

**Figure 1:**
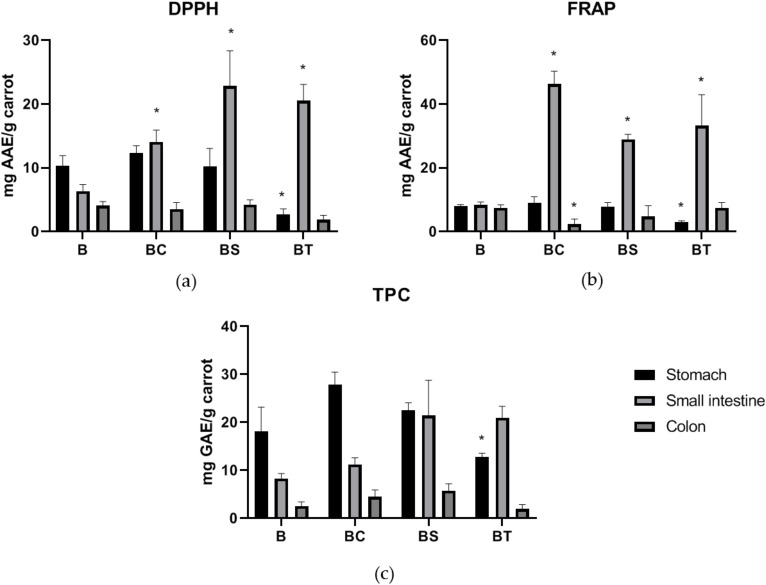
Bioaccessible phytochemicals were increased when associated with lipids. DPPH (**a**), FRAP (**b**), and total phenolic content (**c**) of carrot control and different carrot-oil mixtures from gastric, small intestinal and colonic fractions. Abbreviations: B (black carrot only), BS (black carrot and sunflower oil mixture), BT (black carrot and tallow mixture), and BC (black carrot and coconut oil mixture). * Significant differences at *p* < 0.05 compared to control (B) were highlighted. No significant differences were shown among different types of oil treatments. Results of DPPH and FRAP were expressed as mg ascorbic acid equivalent (AAE) per g of carrot on dry weight basis, and results of TPC were expressed as mg gallic acid equivalent (GAE) per g of carrot on dry weight basis.

Overall increases in bioaccessible antioxidant capacity (DPPH and FRAP) and total phenolic contents were observed in lipid treatments and in line with the results of polyphenols measurements. Gastric fractions showed no impact from presence of lipids except for the tallow group due to the solid state of mixture (Figure 1). During the intestinal digestion, oil groups showed significantly higher DPPH values (*p* < 0.05) compared to the carrot control (Figure 1a) but were not dependent on oil composition as no significantly different DPPH values of the overall antioxidant compounds were shown among different lipids treatments. However, the DPPH assay also takes other antioxidant compounds such as the phenolic acids in glycolysed forms, flavonoids, and ascorbic acids contained in black carrots into account which may explain this result [16,50]. Decreased DPPH activities were obtained in the colonic fraction, indicating the decomposition of most antioxidants related compounds. Strong positive correlations exist between DPPH and FRAP assays confirming these results [16]. It was noticeable that BC in colonic fraction exhibited statistically lower FRAP activity than the carrot control group. This could be related to the shift of gut microbiome during fermentation with coconut treatment (Figure 3), which would result in decreased bacterial activity and lower level of metabolites with reducing capability. In terms of TPC, a range of 18.1 to 31.9 mg gallic acid equivalents (GAE)/g carrot on dry weight (DW) basis were observed at gastric stage with significant lower value in BT group (Figure 1c). No statistical differences were seen at intestinal stages, and this was probably due to the reducing compounds other than antioxidants such as reducing sugars, since Folin–Ciocalteu assay does not specify to antioxidants only but any reducing substance [51]. Thus, the antioxidant assays largely reflected the LC results above and confirmed that inclusion of lipids help increase bioaccessible bioactivity during in vitro digestion.

**Figure 2:**
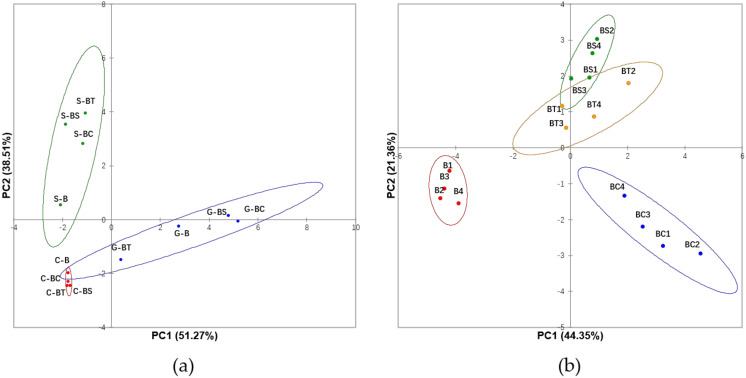
Bioaccessibility of polyphenols and antioxidant measures during digestion of black carrots are explained by the inclusion of lipids. Principal component analysis (PCA) of the dataset of individual polyphenols and phytochemical bioactivities (DPPH, FRAP, and TPC) dataset. Panel (**a**) shows the separation of gastric, small intestinal, and colonic digestive fluids. Panel (**b**) shows the separation of different carrot and oil combinations. Different groups are denoted by colours as shown in the legend. Abbreviations: G (gastric fraction), S (small intestinal fraction), and C (colonic fraction); B (black carrot only), BS (black carrot and sunflower oil mixture), BT (black carrot and tallow mixture), and BC (black carrot and coconut oil mixture).

The effect of the type of lipids on black carrot polyphenol digestion at different digestive stages was considered by a principal component analysis (PCA) of the dataset of the HPLC-DAD quantified individual polyphenols and phytochemical bioactivities (DPPH, FRAP and TPC; Figure 2a). Gastric, small intestinal and colonic fractions could be well separated with 89.8% variability explained with the two principal components and suggested significant roles of intestinal lipid protection and colonic microbiome metabolism of the antioxidant compounds. Figure 2b shows the impact of the different lipid interferences with 65.7% variability explained. To eliminate the impact of tallow solidification in the gastric phase, only small intestinal and colonic fractions datasets were used. The three lipid treatments were separated from the carrot control, indicating that lipids impact on the digestion of bioactive compounds. BS and BT groups were very similar, while BC samples were separated in the PCA space, suggesting a significant role of coconut oil in protecting polyphenols from degradation.

### 3.3. The Carrot-Oil Matrix Modulates the Gut Microbiota and Affects Short Chain Fatty Acid Production

**Figure 3:**
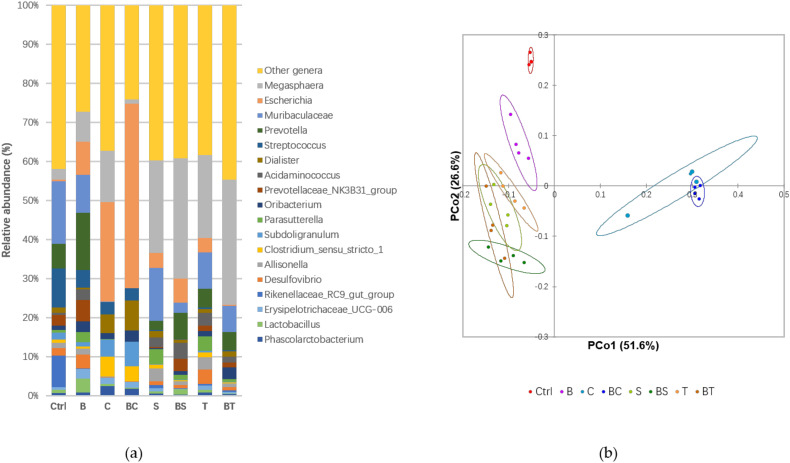
Microbial diversity and abundance were altered by inclusion of lipids in the model diet. Relative abundance of most abundant (>1%) microbial genera (**a**) and weighted UniFrac-principal coordinate analysis (PCoA) analysis of beta diversity among all profiled samples (**b**). Different groups are denoted by colours as shown in the legend. Abbreviations: Ctrl (negative control with faeces only), B (black carrot only), C (coconut oil), BC (black carrot and coconut oil mixture), S (sunflower oil), BS (black carrot and sunflower oil mixture), T (tallow), and BT (black carrot with tallow mixture).

In general, dietary fats are degraded and absorbed in small intestine, however, it has been reported that a portion of ingested fats would enter the human colon and are metabolised by colonic bacteria [8]. To test the interference of lipids on adaptation of the gut microbiota when treated with a digested black carrot matrix, 16S rRNA sequencing was carried out on the faeces samples after 20 h in vitro fermentation. *Megasphaera, Escherichia, Muribaculaceae, Prevotella,* and *Streptococcus* were the most abundant genera (Figure 3a). Significant differences were found between these major genera between the different treatment groups (*p* < 0.0001) (Table 3). It should be noted that this test aims to give a view of the role of gut microbiome on phenol-lipids matrix digestion. In vivo experiments with larger sample size would be required to test more accurate gut microbiome composition changes.

Presence of black carrots changed the abundance and composition of the microbial population and was different to the control (Ctrl) (Figure 3b). The shift in microbial diversity was most likely promoted by dietary fibre and any bound polyphenols that escaped the small intestinal digestion. Beneficial bacteria including *Prevotella*, *Prevotellaceae* NK3B31 *group*, and *Lactobacillus* had higher relative abundance in black carrot diets, while genera such as *Streptococcus* were suppressed (Figure 3a and Table 3). Both *Prevotella* and *Prevotellaceae* NK3B31 *group* were associated with pectin metabolism [52,53]. The metabolism of anthocyanins might promote the growth of *Lactobacillus* [54]. Generally, our results verified that intake of polyphenol-rich dietary polysaccharides shifted microbial diversity to include genera with beneficial consequences.

Lipid preparations played a significant role in modulating the gut microbiota and were largely unaffected by presence of carrots as the carrot-lipid mixed samples closely clustered with the corresponding lipid controls (Figure 3b). The type of lipid was important as seen by the separation of coconut positive control (C) and BC groups from other treatments, while much more clustered and similar microbiome composition were shown between sunflower (S and BS) and tallow (T and BT) groups (Figure 3a,b). *Escherichia* significantly increased (*p* < 0.002) in coconut-containing treatments (Figure 3a and Table 3). Increased *Escherichia* is an indicator of ecosystem disturbance of intestinal microbiota, which is linked with intestinal diseases like constipation and diarrhoea [55]. Zentek, et al. [56] showed that supplementing with the MCFAs capric acid (C10:0), lauric acid (C12:0), and caprylic acid (C8:0) enriched colonic pathogenic *Escherichia* in an in vivo study in piglets. On the other hand, sunflower oil and beef tallow resulted in reduced relative abundance in *Streptococcus* (Table 3). Both long-chain PUFA (especially linoleic acid) and SFA (stearic acid and palmitic acid) have been reported with antibacterial activity against *Streptococcus* [57,58].

Inclusion of coconut oil decreased SCFA production (Table 3; C and BC). Coconut oil contains lauric acid with antibacterial activity [59], and so the low SCFA production of coconut treatment could be a result of the inhibition of the colonic bacteria. Addition of black carrots promoted total SCFAs production compared to the Ctrl as a result of microbial metabolism of the dietary fibre, majorly cellulose and pectin, in black carrot [17]. SCFAs production was not significantly increased by the inclusion of black carrots and consistent with small changes in black carrot inclusion to microbial diversity and composition (Figure 3a,b). Similar results were obtained in our previous in vivo study using a pig model [11]. Inclusion of fat in the diet can decrease colonic microbial cellulolytic and pectinolytic activities [60] and ameliorate any microbial metabolism of dietary fibre. The destruction of the plant cell wall of black carrots during sample preparation before submission to the simulated digestion could contribute to a decreased metabolic rate, as colonic bacteria favour the local microenvironment provided by the cell junctions within large cell clusters compared to exposed cell surfaces for SCFA production [61]. Among the SCFAs, butyrate is an important beneficial SCFA as an energy source for non-transformed colon epithelial cells and can inhibit proliferation of colon cancer cells [62] and is linked to the positive effects of long-term intake of plant-based food rich in dietary fibres [63]. Butyrate production was the highest with sunflower oil (S and BS) with 34.2 mmol/L (Table 3). A study on in vitro colonic fermentation of nuts showed that nuts rich in PUFAs significantly increased the butyrate ratio to the total SCFAs production [64]. Here, the increased butyrate production verified the beneficial effects from PUFAs contained in the sunflower oil.

Overall, the results from this study suggested inclusion of all the three types of lipids increased bioaccessibility of polyphenols during simulated digestion, with coconut oil showing the highest protective effect. At the same time, however, colonic metabolism of coconut oil resulted in increased health detrimental bacteria *Escherichia* and suppressed the production of beneficial colonic SCFAs which are associated with positive cardiovascular effects [65]. Although coconut oil showed the potential to promote absorption of antioxidative polyphenols, it also has been related with increased serum triglycerides and low density lipoproteins (LDL), which are associated with higher risks of cardiovascular diseases [66]. The exact health impact of coconut oil on cardiovascular health remains unclear, and larger clinical intervention studies are required [67]. Our study suggests that the effects of coconut oil on health, especially on cardiovascular health should be assessed with caution. Sunflower oil, on the other hand, also showed protective effects on polyphenols, and it reduced the relative abundance of *Streptococcus,* which is associated with multiple diseases including diarrhoea and obesity [68]. Meanwhile, inclusion of sunflower oil increased butyrate as health beneficial SCFA. PUFA, as the major composition of sunflower oil, has been related with cardioprotective and anti-inflammatory effects [69]. Therefore, without considering effects from compounds other than lipids and polyphenols, from the perspective of promoting polyphenols bioaccessibility and positive gut microbiome adaptation, consuming polyphenol-rich dietary foods coupled with PUFA could be a healthy approach to potentially improve polyphenols absorption with lowest negative effects. Further in vivo experiments with larger sample size and human as subjects are expected to verify this conclusion.

## 4. Conclusions

This study reports the effects of dietary lipids on the bioaccessibility of black carrot polyphenols and microbial diversity in a simulated in vitro digestion and fermentation. Lipids promoted the bioaccessibility of both anthocyanins and phenolic acids during small intestinal digestion, since polyphenol-lipid interactions enhanced the stability of the polyphenols. Coconut oil showed the highest protection of anthocyanins due in part to the medium-chain fatty acids in the coconut oil. Anthocyanins and phenolic acids were largely degraded during colonic fermentation. Inclusion of dietary carrots modulated the gut microbiota and increased the relative abundances of beneficial bacteria, including *Prevotella*, *Prevotellaceae* NK3B31 *group* and *Lactobacillus,* and reduced the detrimental genera *Streptococcus*. Coconut oil appeared to alter microbial growth in the simulated digestion and fermentation with an increase in the abundance of *Escherichia* and suppressed the production of SCFAs. Inclusion of sunflower oil and tallow reduced relative abundance in health detrimental *Streptococcus*. Sunflower oil improved the production of butyrate, potentially due to the presence of PUFAs. The results show that the impact of polyphenols in the digestive tract should be considered in the context of other components of the diet, particularly the lipid component. Coupling polyphenol-rich foods with appropriate amount of PUFA-rich oil could be a beneficial approach to potentially increase polyphenols absorption.

## Figures and Tables

**Table 1:** Fatty acid composition of dietary lipid preparations [12].

Fatty Acid (%)	Coconut Oil	Sunflower Oil	Beef Tallow
C8	7.0	-	-
C10	8.0	-	-
C12	48.0	-	-
C14	16.0	-	3.0
C16	9.5	5.0	26.0
C16:1	-	-	3.0
C18	-	6.0	14.0
C18:1	6.5	30.0	47.0
C18:2	-	59.0	3.0
C18:3	-	-	1.0

**Table 2:** Quantity of individual polyphenols in the black carrot and oil combinations at different digestive compartments (ug/g black carrot DW).

**Identified**	**R**	**Gastric Fraction**			
**Compound**	**value**	**B (BA%)**	**BC (BA%)**	**BS (BA%)**	**BT (BA%)**
Cy 3-xylglcgal	110.9	18.1^b^ (16.3%)	42.2^a^ (38.1%)	45.0^a^ (40.6%)	10.1^b^ (9.10%)
Cy 3-xylgal	850.3	64.1^bc^ (7.50%)	209.5^a^ (24.6%)	194.7^ab^ (22.9%)	48.8^c^ (5.70%)
Cy 3-xyl (sin) glcgal	126.4	39.6^a^ (31.3%)	51.0^a^ (40.3%)	45.7^a^ (36.1%)	8.50^b^ (6.7%)
Cy 3-xyl (fer) glcgal	1483.8	711.6^a^ (48.0%)	823.9^a^ (55.5%)	732.6^a^ (49.4%)	140.5^b^ (9.5%)
Cy 3-xyl (cmr) glcgal	55.6	26.6^ab^ (47.9%)	39.9^a^ (71.7%)	32.5^a^ (58.4%)	6.90^b^ (12.3%)
Total anthocyanins	2627	859.9^a^ (32.7%)	1166^a^ (44.4%)	1050^a^ (40.0%)	214.7^b^ (8.20%)
Neochlorogenic acid	996.2	13.0 (1.30%)	17.1 (1.70%)	17.5 (1.80%)	7.60 (0.80%)
Chlorogenic acid	2470.8	1899^ab^ (76.9%)	2303^a^ (93.2%)	2002^a^ (81.0%)	861.7^b^ (34.9%)
Caffeic acid	109.4	63.5^a^ (58.0%)	51^b^ (46.6%)	47.1^b^ (43.0%)	14.3^c^ (13.0%)
Ferulic acid	135.4	55.8^a^ (41.2%)	40.3^b^ (29.8%)	44.3^b^ (32.8%)	13.2^c^ (9.80%)
*p*-coumaric acid	-	-	-	-	-
Total phenolic acids	3712	2031^a^ (54.7%)	2411^a^ (65.0%)	2111^a^ (56.9%)	896.9^b^ (24.2%)
**Identified**	**R**	**Small Intestinal Fraction**			
**compound**	**value**	**B (BA%)**	**BC (BA%)**	**BS (BA%)**	**BT (BA%)**
Cy 3-xylglcgal	110.9	0.25^b^ (0.23%)	1.76^a^ (1.59%)	1.35^a^ (1.22%)	1.70^a^ (1.54%)
Cy 3-xylgal	850.3	0.95^b^ (0.11%)	9.78^a^ (1.15%)	3.68^b^ (0.43%)	3.02^b^ (0.36%)
Cy 3-xyl (sin) glcgal	126.4	0.80^c^ (0.63%)	3.54^a^ (2.8%)	2.84^ab^ (2.24%)	2.09^b^ (1.66%)
Cy 3-xyl (fer) glcgal	1483.8	23.0^c^ (1.55%)	117^a^ (7.9%)	73.3^b^ (4.94%)	69.1^b^ (4.66%)
Cy 3-xyl (cmr) glcgal	55.6	0.84^c^ (1.51%)	3.00^a^ (5.4%)	1.47^bc^ (2.65%)	1.77^b^ (3.18%)
Total anthocyanins	2627	25.8^c^ (0.98%)	135.3^a^ (5.15%)	82.7^b^ (3.15%)	77.7^b^ (2.96%)
Neochlorogenic acid	996.2	7.73^c^ (0.78%)	5.65^c^ (0.57%)	23.7^b^ (2.37%)	39.3^a^ (3.95%)
Chlorogenic acid	2470.8	101.6^b^ (4.1%)	805.6^a^ (32.6%)	599.5^a^ (24.3%)	862.5^a^ (34.9%)
Caffeic acid	109.4	172.7^b^ (157.9%)	202.3^a^ (184.9%)	210.1^a^ (192.1%)	206.8^a^ (189.1%)
Ferulic acid	135.4	247.5^b^ (182.8%)	236.1^b^ (174.4%)	308.2^a^ (227.7%)	246.0^b^ (181.7%)
*p*-coumaric acid	-	144.5^c^ (n.a.)	168.5^b^ (n.a.)	204.0^a^ (n.a.)	178.5^b^ (n.a.)
Total phenolic acids	3712	673.9^b^ (18.2%)	1418^a^ (38.2%)	1346^a^ (36.3%)	1533^a^ (41.3%)
**Identified**	**R**	**Colonic Fraction**			
**compound**	**value**	**B (BA%)**	**BC (BA%)**	**BS (BA%)**	**BT (BA%)**
Cy 3-xylglcgal	110.9	-	-	-	-
Cy 3-xylgal	850.3	-	-	-	-
Cy 3-xyl (sin) glcgal	126.4	-	-	-	-
Cy 3-xyl (fer) glcgal	1483.8	-	-	-	-
Cy 3-xyl (cmr) glcgal	55.6	-	-	-	-
Total anthocyanins	2627	-	-	-	-
Neochlorogenic acid	996.2	-	-	-	-
Chlorogenic acid	2470.8	-	-	-	-
Caffeic acid	109.4	-	-	-	-
Ferulic acid	135.4	-	90.3^a^ (66.7%)	-	-
*p*-coumaric acid	-	-	-	-	-
Total phenolic acids	3712	-	90.3^a^ (2.43%)	-	-

Different letters (a–c) in the same row indicate significant differences at *p* < 0.05 amongst the 4 treatments (B, BC, BS, and BT). Abbreviations: R (raw carrot powder extracts), B (black carrot only), BC (black carrot and coconut oil mixture), BS (black carrot and sunflower oil mixture), BT (black carrot and tallow mixture), and BA% (bioaccessibility%). n.a. (not applicable). Cy-3-xylglcgal (cyanidin 3-xylosyl(glucosyl)galactoside), cy-3-xylgal (cyanidin 3-xylosylgalactoside), cy-3-xyl(sin)glcgal (cyanidin 3-xylosyl(sinapoylglucosyl)galactoside), cy-3-xyl(fer)glcgal (cyanidin 3-xylosyl(feruloylglucosyl)galactoside), cy-3-xyl(cmr)glcgal (cyanidin 3-xylosyl(coumaroylgluco-syl)galactoside).

**Table 3:** Relative abundance (%) of microbiota (>1%) and short chain fatty acid (SCFA) productions (mmol/L) of controls and samples after colonic fermentation.

Genera	Controls				Samples				*p* Value
	Ctrl	C	S	T	B	BC	BS	BT	
*Megasphaera*	2.87^b^	15.2^ab^	31.1^ab^	26.8^ab^	8.39^b^	1.14^b^	44.7^a^	47.0^a^	<0.0001
*Escherichia*	0.39^c^	29.4^b^	5.01^c^	4.81^c^	9.15^c^	47.8^a^	8.91^c^	0.42^c^	<0.0001
*Muribaculaceae*	16.4^a^	0.06^c^	17.9^a^	11.8^ab^	10.6^ab^	0.01^c^	3.83^bc^	9.84^ab^	<0.0001
*Prevotella*	6.61^bc^	0.06^cd^	3.02^bcd^	6.07^bcd^	15.85^a^	0.01^d^	9.61^ab^	6.93^b^	<0.0001
*Streptococcus*	10.3^a^	3.66^bc^	0.38^c^	0.55^c^	4.82^b^	3.18^bc^	0.26^c^	0.29^c^	<0.0001
*Dialister*	1.32^c^	5.50^a^	2.22^bc^	1.24^c^	0.48^c^	7.77^ab^	1.06^c^	2.20^bc^	<0.0001
*Acidaminococcus*	0.63^c^	0.01^c^	3.13^abc^	4.21^ab^	2.95^abc^	-	6.00^a^	2.18^bc^	<0.0001
*Prevotellaceae* NK3B31 *group*	2.71^b^	-	0.39^c^	1.60^bc^	5.93^a^	-	4.64^a^	1.78^bc^	<0.0001
*Oribacterium*	1.13^b^	1.79^ab^	0.33^b^	1.85^ab^	2.99^ab^	2.86^ab^	1.25^b^	4.37^a^	<0.0001
*Parasutterella*	0.82^b^	0.04^b^	4.99^a^	4.68^a^	2.78^ab^	0.01^b^	1.93^ab^	1.07^b^	<0.0001
*Subdoligranulum*	1.79^bc^	5.09^ab^	0.26^c^	0.42^c^	1.20^bc^	6.42^a^	0.21^c^	0.18^c^	<0.0001
*Clostridium sensu stricto 1*	0.84^b^	5.87^a^	1.13^b^	1.61^b^	0.57^b^	3.84^ab^	0.37^b^	0.45^b^	<0.0001
*Allisonella*	1.41^c^	0.11^c^	4.35^a^	4.09^ab^	1.77^bc^	0.07^c^	1.42^c^	1.39^c^	<0.0001
*Desulfovibrio*	1.97^bc^	0.20^c^	1.39^c^	4.69^a^	3.74^ab^	0.06^c^	1.17^c^	1.27^c^	<0.0001
*Rikenellaceae RC9 gut group*	8.27^a^	-	1.07^b^	0.47^b^	0.18^b^	-	0.08^b^	0.50^b^	<0.0001
*Erysipelotrichaceae* UCG-006	0.79	1.93	1.08	1.38	2.62	1.52	0.16	0.58	0.003
*Lactobacillus*	0.78^b^	0.60^b^	0.63^b^	0.76^b^	4.00^a^	0.33^b^	2.09^ab^	0.16^b^	<0.0001
*Phascolarctobacterium*	0.74^b^	2.81^a^	0.74^b^	1.14^ab^	0.83^b^	1.81^ab^	0.39^b^	0.71^b^	<0.0001
Short chain fatty acids									
Acetate	64.3^a^	31.2^d^	32.3^cd^	42.6^bc^	67.5^a^	37.2^bcd^	40.0^bcd^	46.8^b^	<0.0001
Propionate	15.0^c^	4.00^d^	19.7^bc^	16.3^c^	24.1^b^	3.17^d^	23.3^b^	31.4^a^	<0.0001
Butyrate	22.1^c^	3.57^d^	34.5^a^	27.2^bc^	24.5^bc^	2.99^d^	34.1^a^	30.1^ab^	<0.0001
Total SCFAs	101.4^abc^	37.9^d^	86.4^c^	86.1^c^	116.0^a^	43.4^d^	97.3^bc^	108.3^ab^	<0.0001

Different letters (^a^–^d^) in the same row indicate significant differences at *p* < 0.002 compared among 8 different treatments (Ctrl, C, S, T, B, BC, BS and BT). Abbreviations: Ctrl (negative control with faeces only), C (coconut oil), S (sunflower oil), T (tallow), B (black carrot only), BC (black carrot and coconut oil mixture), BS (black carrot and sunflower oil mixture), BT (black carrot with tallow mixture). SCFA (short chain fatty acid).

## Supplementary Materials

The following are available online at https://www.mdpi.com/2076-3921/9/8/762/s1, Figure S1: HPLC-DAD profile of anthocyanins and phenolic acids.
