# Supplementary material for: Dietary Lipids Influence Bioaccessibility of Polyphenols from Black Carrots and Affect Microbial Diversity under Simulated Gastrointestinal Digestion
Source: Antioxidants (Basel). 2020 Aug 17;9(8):762. doi: 10.3390/antiox9080762 (PMC7464840; doi:10.3390/antiox9080762)
Supplement: Supplementary file 1 [file antioxidants-09-00762-s001.pdf]

Supplemental material

Dietary Lipids Influence Bioaccessibility of Polyphenols from Black Carrots and Affect Microbial Diversity under Simulated Gastrointestinal Digestion

Chunhe Gu [1], Hafiz A. R. Suleria [1], Frank R. Dunshea [1,2] and Kate Howell [1,*]

1. School of Agriculture and Food, Faculty of Veterinary and Agricultural Sciences, The University of Melbourne, Parkville 3010, VIC, Australia; chunheg@student.unimelb.edu.au (C.G.); hafiz.suleria@unimelb.edu.au (H.A.R.S.); fdunshea@unimelb.edu.au (F.R.D.)
2. Faculty of Biological Sciences, The University of Leeds, Leeds LS2 9JT, UK

* Correspondence: khowell@unimelb.edu.au; Tel.: +61-470-439-67

Received: 22 July 2020; Accepted: 14 August 2020; Published: date

Abstract: The bioaccessibility and activity of polyphenols is dependent on their structure and entrapment in the food matrix. While dietary lipids are known to transit into the colon, the impact of different lipids on the microbiome, and their interactions with dietary polyphenols are largely unknown. Here, we investigated the effect of dietary lipids on the bioaccessibility of polyphenols from purple/black carrots and adaptation of the gut microbiome in a simulated *in vitro* digestion-fermentation. Coconut oil, sunflower oil, and beef tallow were selected to represent common dietary sources of medium-chain fatty acids (MCFAs), long-chain polyunsaturated fatty acids (PUFAs), and long-chain polysaturated fatty acids (SFAs), respectively. All lipids promoted the bioaccessibility of both anthocyanins and phenolic acids during intestinal digestion with coconut oil exhibiting the greatest protection of anthocyanins. Similar trends were shown in antioxidant assays (2,2-Diphenyl-1-pricrylhydrazyl (DPPH), ferric reducing ability (FRAP), and total phenolic content (TPC)) with higher phytochemical bioactivities observed with the addition of dietary lipids. Most bioactive polyphenols were decomposed during colonic fermentation. Black carrot modulated diversity and composition of a simulated gut microbiome. Dramatic shifts in gut microbiome were caused by coconut oil. Inclusion of sunflower oil improved the production of butyrate, potentially due to the presence of PUFAs. The results show that the impact of polyphenols in the digestive tract should be considered in the context of other components of the diet, particularly lipids.

Keywords: anthocyanin; phenolic acid; antioxidant capacity; medium chain fatty acids; short chain fatty acids; gut microbiome

---

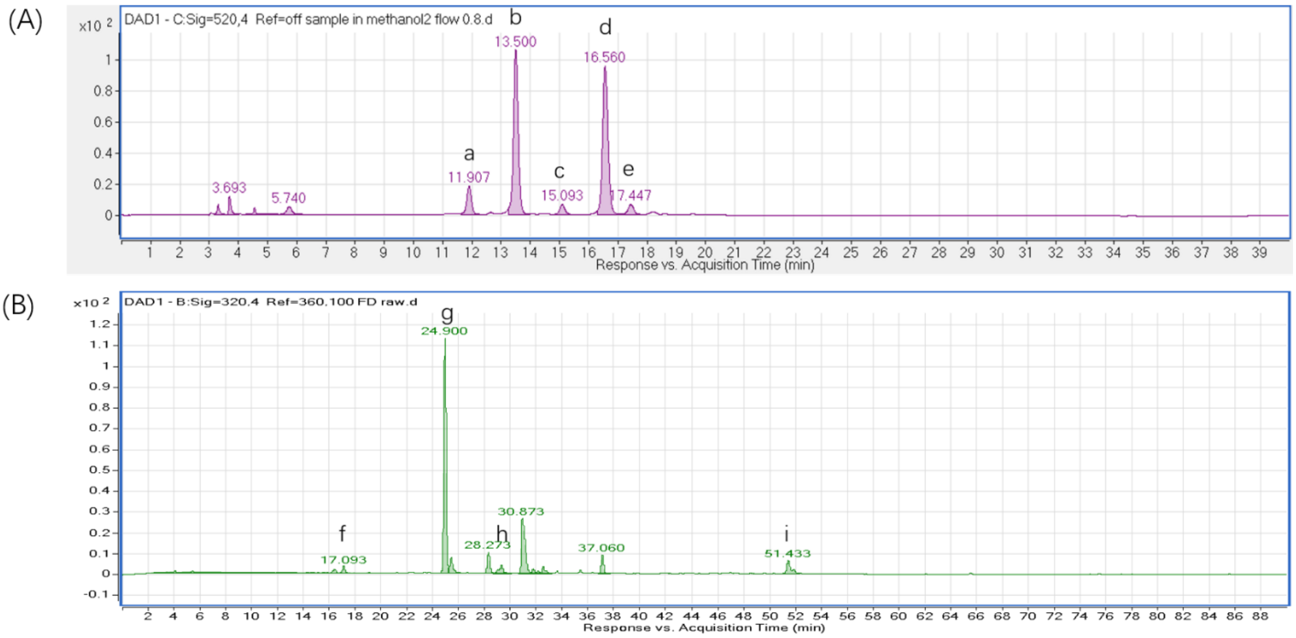

Figure S1. HPLC-DAD profile of anthocyanins (A) at 520 nm and phenolic acids (B) at 320 nm of raw black carrot extracts. Peaks: (a) cyanidin 3-xylosyl-(glucosyl)galactoside, (b) cyanidin 3-xylosylgalactoside, (c) cyanidin 3-xylosyl-(sinapoylglucosyl)galactoside, (d) cyanidin 3-xylosyl(feruloylglucosyl)galactoside, (e) cyanidin 3-xylosyl-(coumaroylglucosyl)galactoside, (f) neo-chlorogenic acid, (g) chlorogenic acid, (h) caffeic acid, (i) ferulic acid
